# Supplementary material for: The Diverse Salt-Stress Response of Arabidopsis ctr1-1 and ein2-1 Ethylene Signaling Mutants Is Linked to Altered Root Auxin Homeostasis
Source: Plants (Basel). 2021 Feb 27;10(3):452. doi: 10.3390/plants10030452 (PMC7997360; doi:10.3390/plants10030452)
Supplement: Supplementary file 1 [file plants-10-00452-s001.pdf]

Supplementary materials

The Diverse Salt Stress Response of Arabidopsis *ctr1-1* and *ein2-1* Ethylene Signaling Mutants is Linked to Altered Root Auxin Homeostasis

Irina I. Vaseva [1,*], Kiril Mishev [1], Thomas Depaepe [2], Valya Vassileva [1] and Dominique Van Der Straeten [2]

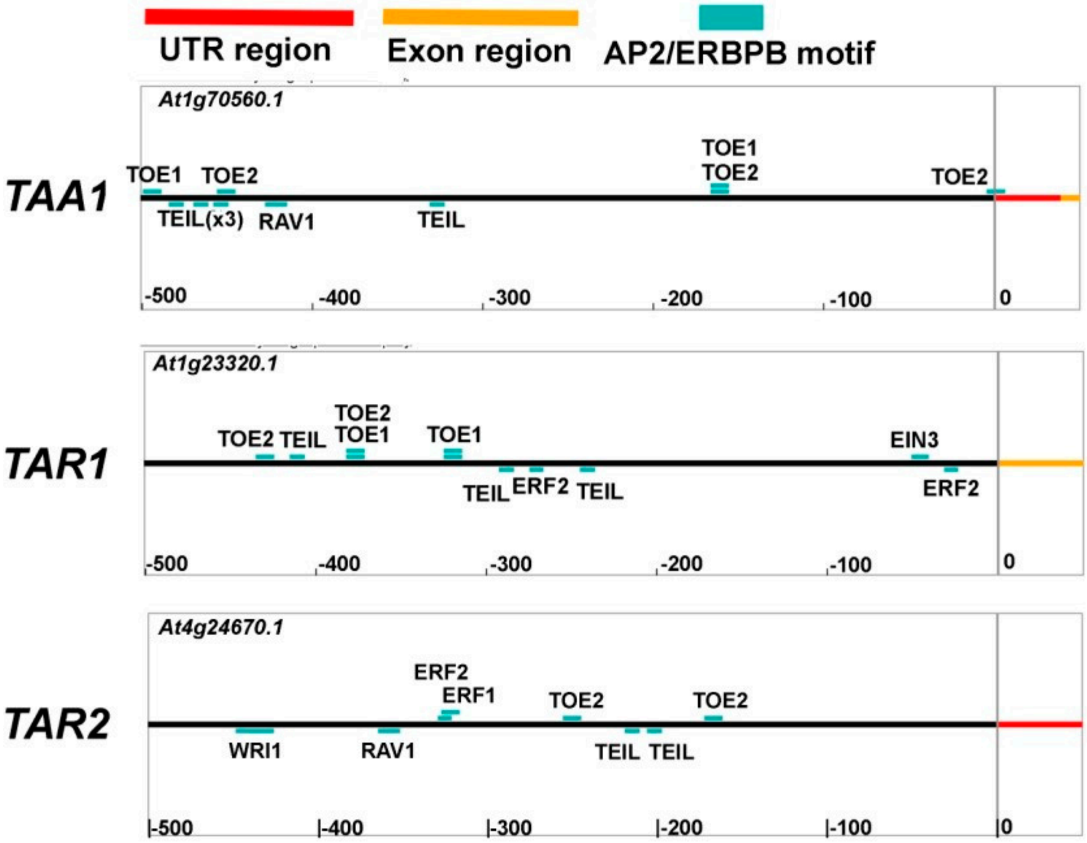

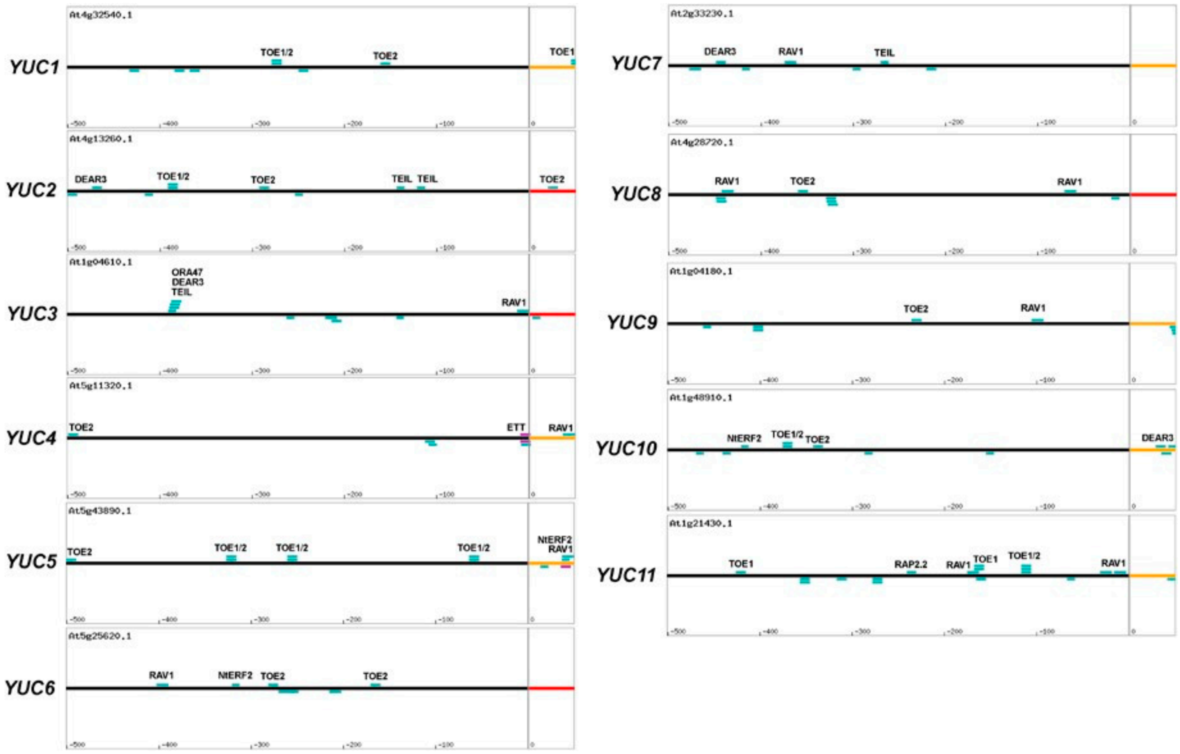

Figure S1. AthaMap analyses of promoter regions (−500 to +50 b.p.) of the genes coding for TAA1, TAR1, TAR2, andYUCs (1-11).

Table S1. TF DeCON in silico screen of the auxin Trp-dependent biosynthesis TAA1 (At1g70560), TAR1 (At1g23320), TAR2 (At4g24670) and YUC1-11 (resp. AT4G32540, AT4G13260, AT1G04610, AT5G11320, AT5G43890, AT5G25620, AT2G33230, AT4G28720.

TF ID	Family	Gene name	Genome count	Genome ratio	Query count	Query ratio	logFC	P valalue	adj.P value
AT1G46768	AP2-EREBP	**RAP2.1**	1889	0.068306	5	0.357143	2.386419	0.001757	0.018726
AT4G06746	AP2-EREBP	**RAP2.9**	2105	0.076116	5	0.357143	2.230221	0.002841	0.023911
AT5G25810	AP2-EREBP	**TINY**	1602	0.057928	4	0.285714	2.302239	0.007028	0.036683

AT1G04180, AT1G48910, AT1G21430) using public database. The data analysis was done by setting the maximum *p*-value at 0.05 and the logFC filter at 2 to narrow down the displayed results

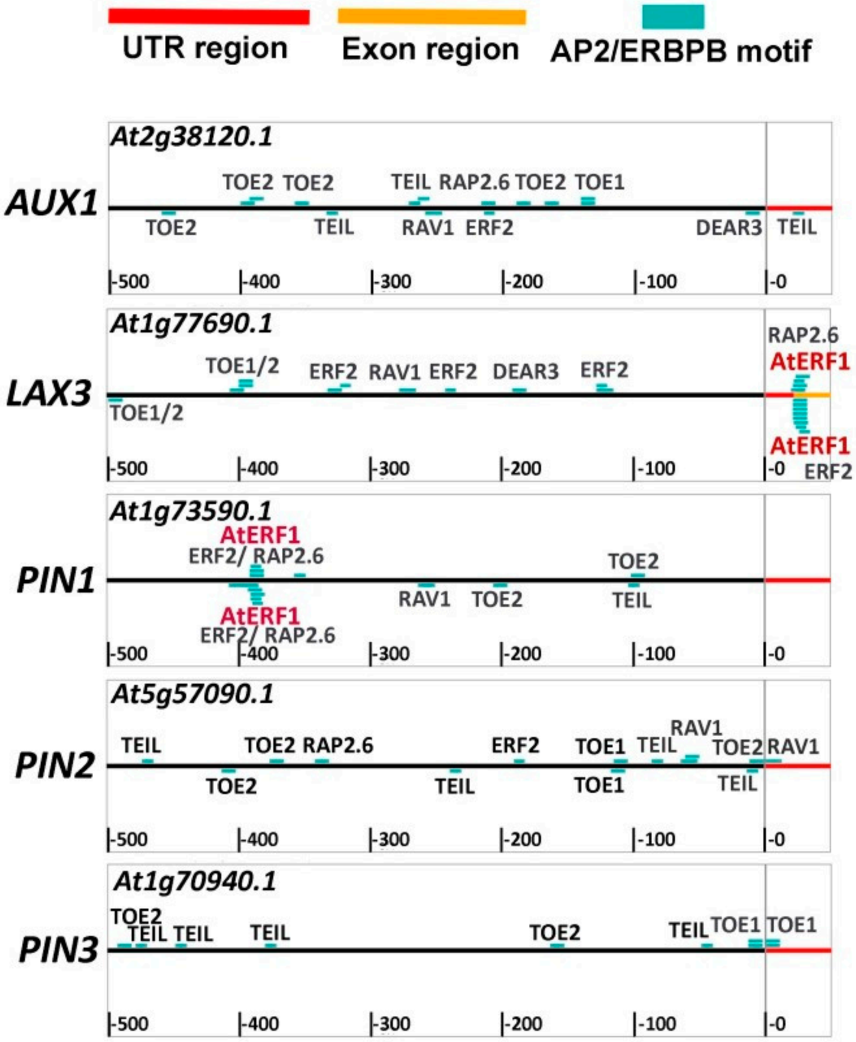

Figure S2. AthaMap analyses of promoter regions (−500 to +50 b.p.) of the auxin transporter genes *AUX1*, *LAX3*, *PIN1*, *PIN2*, and *PIN3*.

Table S2. TF DeCON in silico screen of the auxin transport genes PIN1-8 (resp. AT1G73590, AT5G57090, AT1G70940, AT2G01420, AT5G16530, AT1G77110, AT1G23080, AT5G15100), AUX1/LAX1/ 2/ 3 (AT2G38120, AT5G01240, AT2G21050, AT1G77690) and ABCB1/4/19.

TF ID	Family	Gene name	Genome count	Genome ratio	Query count	Query ratio	logFC	P valalue	adj.P value
AT1G46768	AP2-EREBP	**RAP2.1**	1889	0.068305912	6	0.4	2.549917643	**0.000294507**	0.025499675
AT1G44830	AP2-EREBP	**ERF014**	1398	0.050551437	5	0.333333333	2.721141586	**0.000642796**	0.030885482
AT5G25810	AP2-EREBP	**TINY**	1602	0.057928042	5	0.333333333	2.524631787	**0.001193024**	0.0341608

The data analysis was done by setting the maximum *p*-value at 0.05 and the logFC filter at 2 to narrow down the displayed results.

Table S3. Primers used in the qRT-PCR analyses.

Gene Name	Locus	Amplicon Length (b.p.)	Forward Primer (5′-3′)	Reverse Primer (5′-3′)
AUX1	AT2G38120	151	GAGGTCACGCGGTTACTGTT	GAGAGAAAGCGTTGGAGTGG
LAX3	AT1G77690	185	CACAACTTGGGATGATGTCG	AATGTTTCCCCAACAATCCA
PIN1	AT1G73590	224	TAAGGTGATGCCACCAACA A	GCCATGAACAACCCAAGACT
PIN2	AT5G57090	235	CTTTCTTTGGCAGGCGTTTA	ACTGCTCGGAGATGAGAAGC
PIN3	AT1G70940	209	ATCTTCTCACCCGACCAATG	GATGCTCCACTCGAGGCTAC
ACTIN2	AT3G18780	67	CTTGCACCAAGCAGCATGA A	CCGATCCAGACACTGTACTTC CTT
EF1ALPHA	AT5G60390	151	AGCACGCTCTTCTTGCTTTC	GGGTTGTATCCGACCTTCTTC
